# Pregnancy-Related Acute Kidney Injury in Preeclampsia: Risk Factors and Renal Outcomes

**DOI:** 10.1161/HYPERTENSIONAHA.119.13089

**Published:** 2019-09-30

**Authors:** Frances I. Conti-Ramsden, Hannah L. Nathan, Annemarie De greeff, David R. Hall, Paul T. Seed, Lucy C. Chappell, Andrew H. Shennan, K. Bramham

**Affiliations:** 1 From the Department of Women and Children’s Health, King’s College London, United Kingdom; (F.I.C-R., H.L.N., P.T.S., L.C.C., A.H.S., K.B.); 2 Accuracy Assessed Medical Devices CC, Diamond, South Africa (A.D.g.); 3 Department of Obstetrics and Gynaecology, Stellenbosch University and Tygerberg Hospital, Cape Town, South Africa (D.R.H.).

**Keywords:** acute kidney injury, creatinine, preeclampsia, pregnancy, renal insufficiency, chronic

## Abstract

Preeclampsia is a common cause of acute kidney injury (AKI) in low- and middle-income countries, but AKI incidence in preeclampsia, its risk factors, and renal outcomes are unknown. A prospective observational multicenter study of women admitted with preeclampsia in South Africa was conducted. Creatinine concentrations were extracted from national laboratory databases for women with maximum creatinine of ≥90 μmol/L (≥1.02 mg/dL). Renal injury and recovery were defined by Kidney Disease Improving Global Outcomes creatinine criteria. Predefined risk factors, maternal outcomes, and neonatal outcomes were compared between AKI stages. Of 1547 women admitted with preeclampsia 237 (15.3%) met AKI criteria: 6.9% (n=107) stage 1, 4.3% (n=67) stage 2, and 4.1% (n=63) stage 3. There was a higher risk of maternal death (n=7; relative risk, 4.3; 95% CI, 1.6–11.4) and stillbirth (n=80; relative risk, 2.2; 95% CI, 1.8–2.8) in women with AKI compared with those without. Perinatal mortality was also increased (89 of 240; 37.1%). Hypertension in a previous pregnancy was the strongest predictor of AKI stage 2 or 3 (odds ratio, 2.24; 95% CI, 1.21–4.17). Renal recovery rate reduced with increasing AKI stage. A third of surviving women (76 of 230 [33.0%]) had not recovered baseline renal function by discharge. Approximately half (39 of 76; 51.3%) of these women had no further creatinine testing post-discharge. In summary, AKI was common in women with preeclampsia and had high rates of associated maternal and perinatal mortality. Only two-thirds of women had confirmed renal recovery. History of a previous hypertensive pregnancy was an important risk factor.

## Abstract 2

Supplemental Digital Content is available in the text.

The global incidence of pregnancy-related acute kidney injury (AKI) has reduced over recent decades because of improvements in reproductive health care.^1–4^ Pregnancy-related AKI remains a common cause for requiring dialysis in low- and middle-income countries^3,5,6^ and is associated with high rates of maternal and neonatal morbidity and mortality.^5,7^ There is limited understanding of underlying risk factors in these settings to enable appropriate triage and targeting of scare resources. In addition, currently published studies are predominantly retrospective, are limited by diverse definitions of AKI, and few report incidence according to Kidney Disease Improving Global Outcomes (KDIGO) criteria.^6^

Outside of pregnancy, there is increasing evidence that AKI is a risk factor for chronic kidney disease (CKD),^8,9^ and a recent meta-analysis of population studies has reported that CKD is common in low-income countries. Nearly twice as many women of reproductive age in low-income countries have CKD compared with high-income countries (9.0% versus 5.9%, respectively),^10^ but the relationship between pregnancy-related AKI and subsequent CKD in low- and middle-income countries is unknown.

Worldwide, hypertensive disorders of pregnancy are the most common cause of pregnancy-related AKI.^5,7,11,12^ The Community blood pressure monitoring in Rural Africa and Asia: Detection of underLying preEclampsia and shock (CRADLE) 2 study was a prospective observational cohort of pregnant or postpartum women admitted with preeclampsia in 3 state tertiary maternity units in South Africa (January 2015 to May 2016). National laboratory databases in South Africa hold all routinely collected laboratory data thus facilitating assessment of renal recovery after AKI.

The aims of this secondary analysis of CRADLE 2 were to examine the incidence of pregnancy-related AKI according to KDIGO AKI criteria and to identify risk factors and report renal outcomes of women with preeclampsia who developed such AKI.

## Methods

The data that support the findings of this study are available from the corresponding author on reasonable request. The CRADLE 2 trial was a prospective observational cohort study of women admitted with preeclampsia at 3 tertiary hospital sites (Groote Schuur Hospital, Tygerberg Hospital, and Kimberley Hospital) in South Africa between January 2015 and May 2016. Any woman with a clinical diagnosis of preeclampsia as determined by their healthcare provider during their admission was included. Baseline and admission characteristics recorded were age, body mass index, parity, gestation at admission, admission systolic BP (SBP) and diastolic blood pressure (DBP), highest SBP during admission, DBP at highest SBP, and admission urine dipstick result (1+ to 3+). Prespecified clinical outcomes were eclampsia, stroke, maximal creatinine ≥90 µmol/L (≥1.02 mg/dL) during admission, and maternal and perinatal death. BP was measured using the Microlife CRADLE Vital Signs Alert—a device validated for use in pregnancy including preeclampsia.^13^ The study was approved by the Ethics Committee at each hospital site: Stellenbosch University Ethics Committee (N14/06068), University of Cape Town Ethics Committees (410/2014), and the University of the Free State Ethics Committee (230408-011); individual written consent was obtained at 2 sites, and institutional-level consent was granted at the third site. The study was conducted in line with the Declarations of Helsinki. The full findings of the study are published in the Journal of Global Health.^14^ The original study was funded by Bill & Melinda Gates Foundation (grant ID: OPP1086183).

Serial creatinine values including any prepregnancy test results up until May 2017 were extracted from national laboratory databases in all women who had a maximum creatinine of ≥90 μmol/L during their admission. Hematological and biochemical test results at time of maximum creatinine of ≥90 μmol/L were also recorded. At one hospital site (Groote Schuur Hospital) where clinical records were available at the time of the study, original clinical notes were reviewed for all women who had a maximum creatinine of ≥90 μmol/L during admission and a random unmatched sample of 100 controls (maximum creatinine <90 μmol/L during admission), with 100 ID numbers selected using the Stata, version 14.2 (StataCorp, College Station, TX), random uniform() command. Candidate risk factors for obstetric AKI were recorded: maternal age, body mass index, gravidity, parity, medical comorbidities—anemia^15^ (hemoglobin <9 g/dL), chronic hypertension (documented history or SBP >140 or diastolic >90 mm Hg before 20 weeks of gestation or taking antihypertensive medication), and HIV status—and documented history of hypertensive disorder in a previous pregnancy. All 3 hospital sites gave ethical approval for this additional data collection as amendments to the original ethics application. Funding for this substudy was provided by the British Maternal Fetal Medicine Society and a Royal College of Obstetricians and Gynaecologists travel award.

The KDIGO serum creatinine criteria were applied to the serial creatinine data to determine AKI stage, renal recovery at discharge, and recovery at follow-up for all women with a maximum creatinine of ≥90 μmol/L during admission for preeclampsia. Baseline creatinine was defined as the single lowest creatinine concentration <90 μmol/L ≤2 years before admission date up until the end of May 2017. Where women had no creatinine value <90 μmol/L recorded, minimum creatinine during admission was used as a proxy for baseline creatinine. Maximal creatinine was defined as the single highest creatinine concentration ≥90 μmol/L during admission. Discharge creatinine was defined as the single lowest creatinine concentration on the last day creatinine was recorded before and including day of discharge. Follow-up creatinine was defined as the single lowest creatinine concentration post-discharge until the end of May 2017. KDIGO stage was calculated as per KDIGO criteria using ratios of maximum creatinine to baseline creatinine or minimum creatinine with a ratio of >1.5, 2, and 3 denoting stages 1, 2, and 3, respectively. A rise in serum creatinine concentration of ≥26 μmol/L in 48 hours during admission also denoted stage 1 AKI, and a maximum of >354 μmol/L was classed as stage 3 AKI. Recovery at discharge or follow-up was determined using ratios of creatinine at discharge and follow-up, respectively, to baseline or minimum creatinine concentration with a ratio of <1.5 denoting recovery.

Ordered logistic regression modeling was used to evaluate the association between baseline demographic and clinical characteristics and development and severity of AKI (outcome considered as ordered categories: no AKI, maximum creatinine ≥90 μmol/L but AKI criteria not met, stage 1 AKI, stage 2 AKI, stage 3 AKI). Logistic regression models and area under the receiver operator curve values with 95% CIs were used to assess correlation between baseline demographic, clinical, and admission characteristics and KDIGO score. Non parametric tests (Kruskal-Wallis test) were used to perform simple comparisons between groups. All data manipulation and analysis was performed in Stata, version 14.2 (StataCorp, College Station, TX).

## Results

### AKI Staging

**Figure 1.**
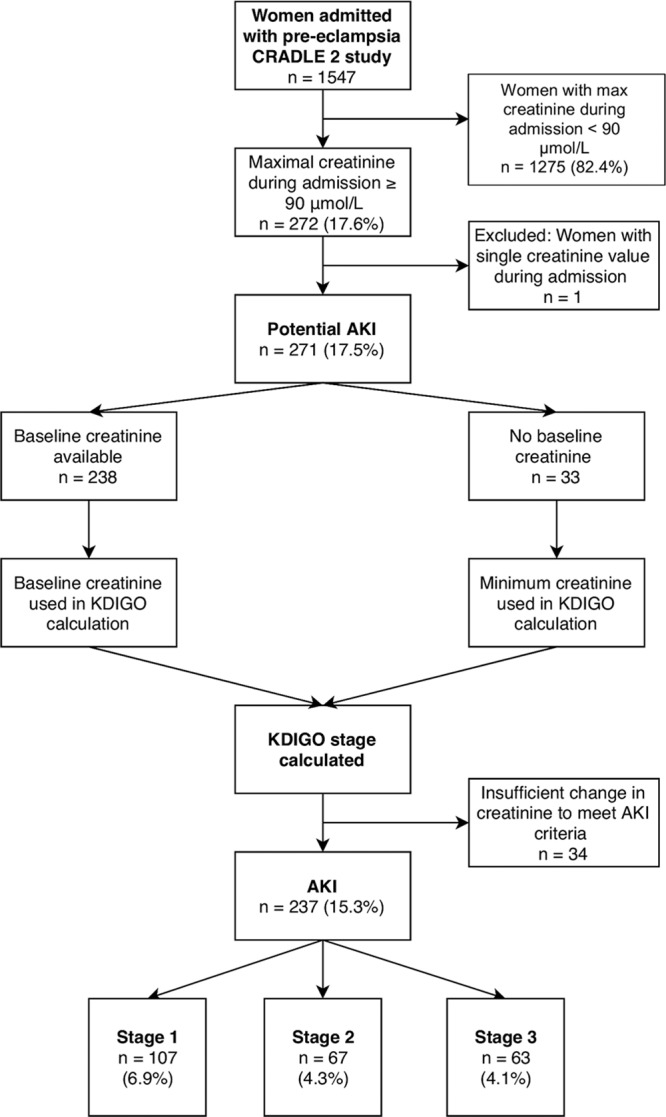
AKI indicates acute kidney injury; CRADLE, Community blood pressure monitoring in Rural Africa and Asia: Detection of underLying preEclampsia and shock; and KDIGO, Kidney Disease Improving Global Outcomes.

One thousand five hundred forty-seven women with preeclampsia were recruited of whom 272 (17.6%) had a maximum creatinine concentration of ≥90 μmol/L (≥1.02 mg/dL) during admission. Of these, 15.3% (n=237) had serial changes in serum creatinine concentration consistent with AKI KDIGO criteria (Figure 1; Table 1). One woman was excluded from the study because she had only a single recorded creatinine concentration on the national laboratory database. Of the 1547 women recruited, 6.9% (n=107) had stage 1 AKI, 4.3% (n=67) had stage 2 AKI, and 4.1% (n=63) had stage 3 AKI. The remaining 35 women had insufficient rise in creatinine concentration to meet criteria for stage 1 AKI, despite a maximum creatinine concentration of ≥90 μmol/L during admission, or had a single creatinine concentration recorded in the national laboratory databases precluding any assessment of dynamic changes. In these 237 women with AKI, creatinine, urea concentrations, and white cell count increased across AKI stages and hemoglobin concentration reduced (S1). Of the 32 women with stage 3 AKI with hospital records available, 4 (12.5%) required dialysis, with median duration of dialysis being 4 days (range, 2–5).

**Table 1.**
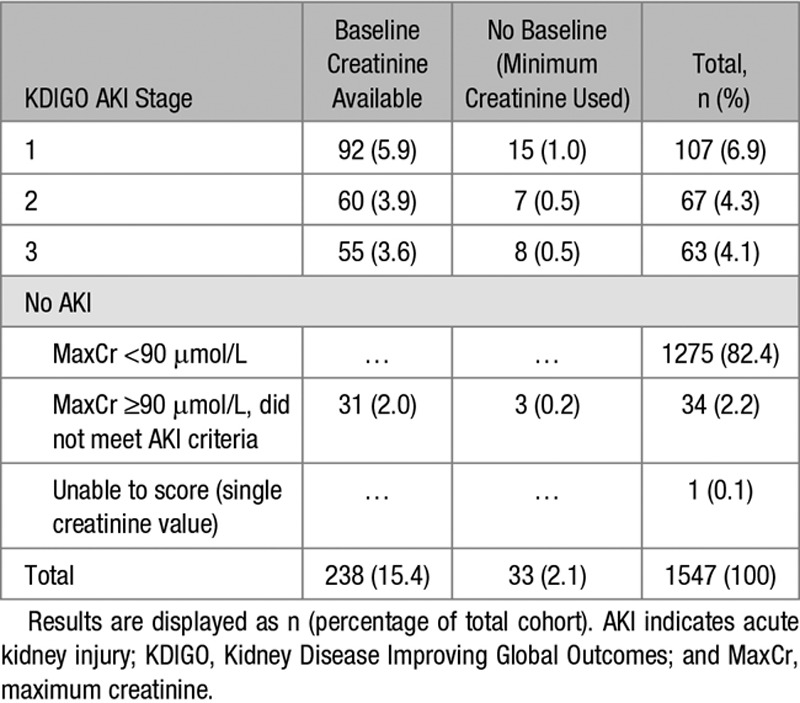
AKI Staging as per Definitions in Box 1

### Maternal and Neonatal Outcomes Associated With AKI

Baseline demographics, admission characteristics and pregnancy, and maternal and neonatal outcomes in the groups are shown in Table 2. Women who developed AKI (stages 1–3) were more likely to die (risk ratio [RR], 4.3; 95% CI, 1.6–11.4; *P*=0.003), have had an eclamptic seizure (RR, 1.7; 95% CI, 1.2–2.4; *P*=0.005), have a stroke (RR, 16.6; 95% CI, 1.7–158.8; *P*=0.015), have received magnesium sulfate (RR, 1.2; 95% CI, 1.1–1.2; *P*<0.001), or be admitted to intensive care (RR, 1.9; 95% CI, 1.6–2.2; *P*<0.001) compared with women who did not develop AKI during admission with preeclampsia. Overall, there were 7 maternal deaths in the women who met KDIGO AKI criteria (7 of 237; 3.0%), with no significant difference between AKI stages (*P*=0.803). However, rates of eclampsia increased with AKI stage (*P* for trend, 0.02; stage 3 versus stage 1 AKI RR, 2.3; 95% CI, 1.1–4.7; *P*=0.02), in parallel with rates of intensive care unit admission (*P* for trend, <0.001; stage 3 versus stage 1 AKI RR, 2.1; 95% CI, 1.5–2.9; *P*<0.001; stage 2 versus stage 1 AKI RR, 1.7; 95% CI, 1.2–2.4; *P*=0.003).

**Table 2.**
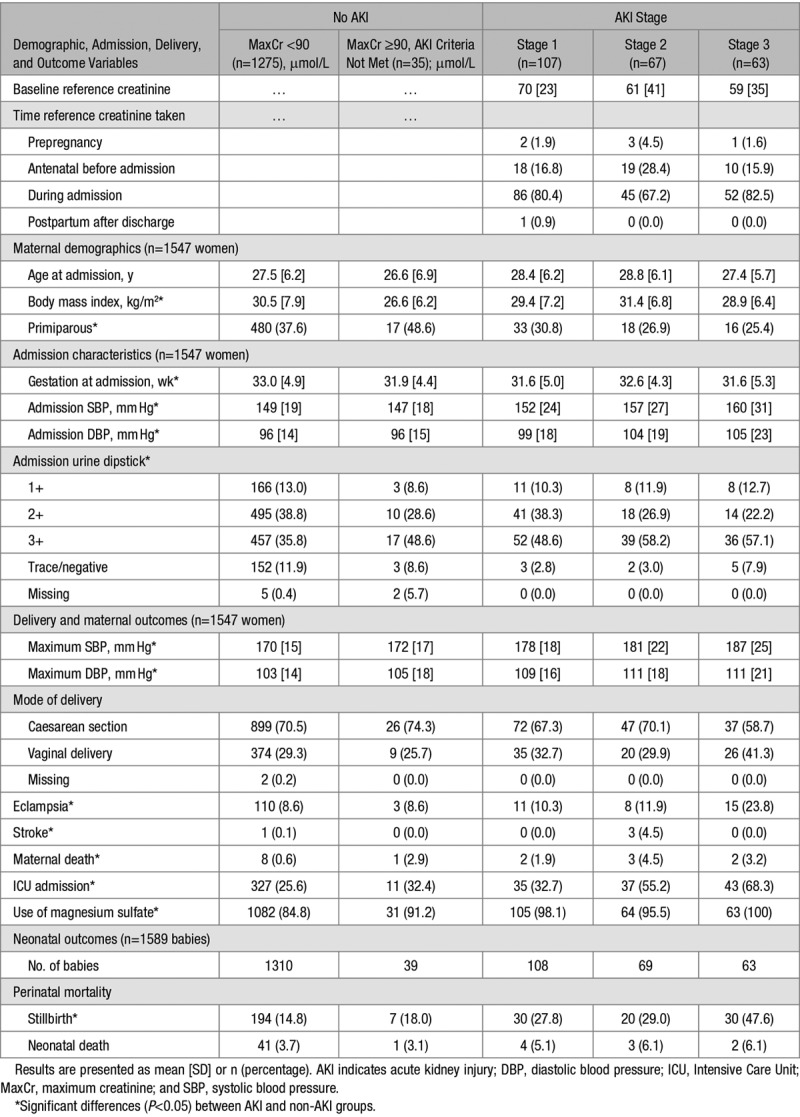
Maternal Demographics, Admission Characteristics, and Delivery, Maternal and Neonatal Outcomes by AKI Category, and AKI Stage as Defined by Kidney Disease Improving Global Outcomes in Women With Preeclampsia

Women who developed AKI were also more likely to experience a stillbirth (RR, 2.2; 95% CI, 1.8–2.8; *P*<0.001) compared with women without AKI. This was unchanged by adjustment for maximum SBP during admission, DBP at maximum SBP, and gestation at admission in models (odds ratio, 3.32; 95% CI, 2.29–4.81; *P*<0.001 [results presented as odds ratio rather than RR as binomial regression models failed to converge]). There were no significant differences in rates of neonatal deaths between women with and without AKI (RR, 1.5; 95% CI, 0.8–3.1; *P*=0.229). Stillbirth rates increased significantly with increasing AKI severity (*P* for trend, 0.01; stage 3 versus stage 1 AKI RR, 1.7; 95% CI, 1.1–2.6; *P*=0.008), and adjustment for maximum SBP during admission, DBP at maximum SBP, and gestation at admission similarly did not alter results (stage 3 versus stage 1 AKI odds ratio, 2.61; 95% CI, 1.29–5.27; *P*=0.007). Overall perinatal mortality in babies born to women with AKI including twin pregnancies was 37.1% (89 of 240).

### Risk Factors for AKI

**Figure 2.**
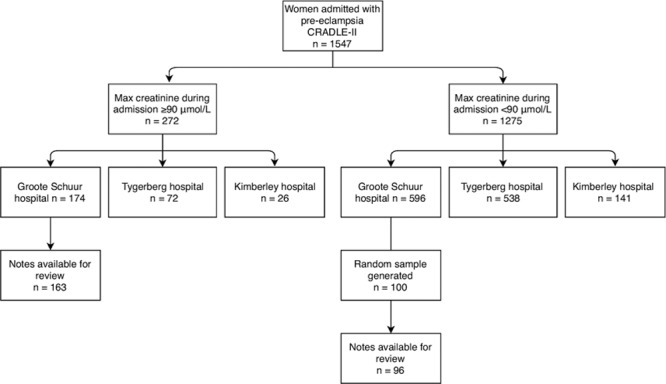
Flow diagram illustrating selection of cases for clinical record review from single hospital site. CRADLE indicates Community blood pressure monitoring in Rural Africa and Asia: Detection of underLying preEclampsia and shock.

Selection for cases and controls at the single hospital site is shown in Figure 2. Original patient files were available for 163 of 174 (94%) cases with maximum creatinine concentration ≥90 µmol/L and 96 of 100 (96%) randomly selected controls (maximum creatinine <90 µmol/L). Maternal demographics and comorbidities by AKI category and stage are shown in Table S2 in the online-only Data Supplement. Results of individual ordered logistic regression for each predefined risk factor are shown in Table 3. Following stepwise ordered logistic regression, only history of hypertensive disorder of pregnancy and maternal age were significant predictors of AKI (Table 3). The analysis was repeated using a cutoff point of stages 2 or more AKI to assess the robustness of this finding, and previous history of hypertensive disorder alone was also predictive of stage 2 or 3 AKI (odds ratio, 2.24; 95% CI, 1.21–4.17; *Z* score, 2.56; *P*=0.011).

**Table 3.**
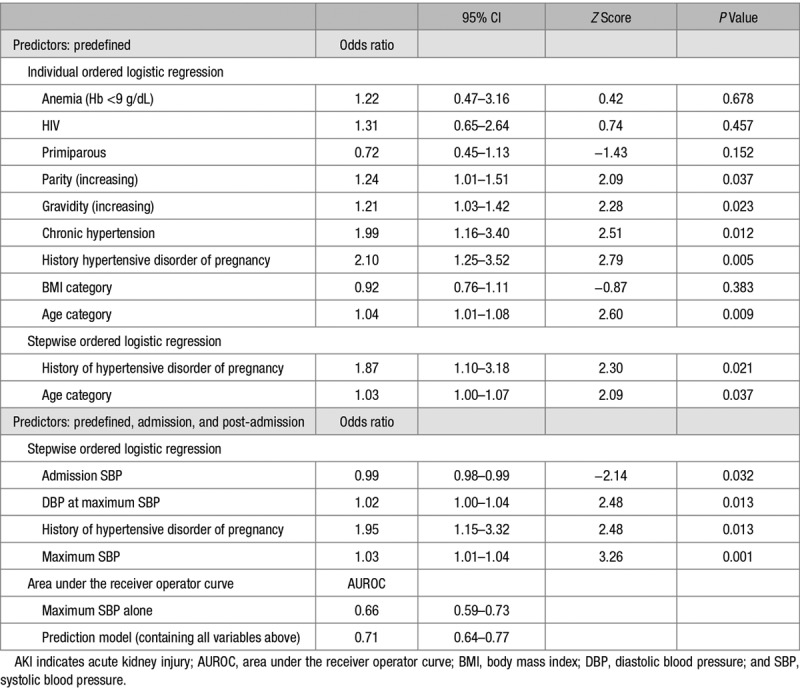
Results of Individual and Stepwise Ordered Logistic Regression and AUROC for Predefined Clinical Predictors, With and Without Admission and Postadmission Variables Included in Models, for Development of AKI and Increasing AKI Severity

When postadmission factors (gestation at admission, admission SBP and DBP, admission urine dipstick findings, and maximum SBP and DBP at maximum DBP) were included in the stepwise logistic regression model with predefined risk factors, history of hypertensive disorder of pregnancy and BP variables remained significant predictors (Table 3). Maximum SBP during admission was the best individual predictor of AKI severity with a comparable area under the receiver operator curve to the adjusted prediction model (Table 3).

### Renal Recovery at Discharge and Follow-Up

Renal recovery at discharge or during follow-up according to AKI stage is shown in Table 4. Overall, 154 of 230 (67.0%) surviving women had recovered from AKI at discharge from hospital. Rate of renal recovery at discharge reduced significantly with increasing stage of AKI; 95 of 105 (90.5%) women with stage 1 AKI while only 38 of 64 (59.3%) women with stage 2 and 21 of 61 (34.4%) with stage 3 AKI had recovered function (*P*<0.01). Of the 76 women who had not recovered renal function at discharge, 39 (51.3%) had no further creatinine concentrations assessed. Of the 37 women with repeat creatinine concentrations post-discharge, 31 (83.8%) had renal recovery and 6 (16.2%) did not. These 6 women had all experienced stage 2 or 3 AKI. For women who recovered renal function after discharge, creatinine testing confirming recovery was taken at a median of 38 (IQR, 17–208) days after discharge. Overall rate of confirmed renal recovery was higher for stage 1 AKI (94.3%), compared with stage 2 (70.3%) and stage 3 (67.2%) AKI. However, many women did not have creatinine concentration repeated after discharge including 23.4% of women with stage 2 and 29.5% of women with stage 3 AKI (Table 4).

**Table 4.**
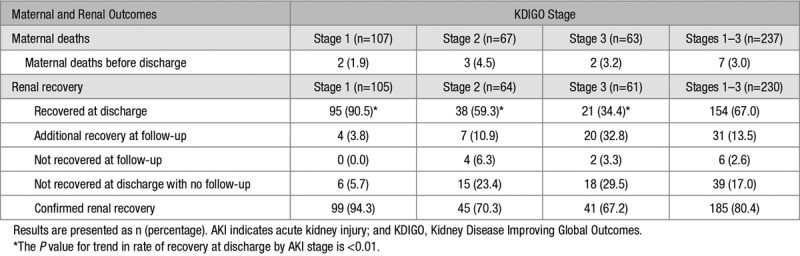
Maternal Deaths and Renal Recovery at Discharge, Follow-Up, and Overall in Surviving Women by AKI Stage

## Discussion

### Principal Findings

Fifteen percent of women admitted to hospital with preeclampsia in this middle-income country cohort had pregnancy-related AKI assessed by KDIGO criteria, with over half of cases being stage 2 or 3 in severity. Maternal death, eclampsia, stroke, and stillbirth rates were higher in these women than those without. A novel finding of this study was the high proportion of women with pregnancy-related AKI with a history of hypertensive disorder in a previous pregnancy, also more frequent with increasing AKI severity. Approximately two-thirds of women had recovered from AKI at discharge, with lower rates of renal recovery with more severe AKI stages. Overall, 2.6% of women had not recovered from AKI on follow-up, all of whom had stage 2 or 3 AKI. However, overall rate of renal recovery in those with repeat creatinine concentration testing was high, although over 50% of women with pregnancy-related AKI who had not recovered renal function at discharge had no repeat testing.

### Strengths and Limitations

The strengths of this study include its large, multicenter, prospective design with comprehensive national laboratory serial creatinine concentration data analysis, which allowed KDIGO staging of AKI and analysis of risk factors and outcomes according to AKI stage. Limitations include all centers being in a single country, which means findings may not be generalizable to other low- and middle-income settings. Furthermore, the diagnosis of preeclampsia was a clinical diagnosis determined by healthcare provider rather than application of validated diagnostic criteria. Lack of follow-up in over half of women who had not recovered baseline renal function from AKI by discharge from hospital impacted on our ability to determine the overall renal outcomes of AKI. In addition, other clinical events that may have affected development and severity of AKI such as hemorrhage, sepsis, nephrotoxin exposure, HIV viral load and fluid, and blood resuscitation, were not captured. Lastly, clinical record review to investigate risk factors for AKI was only possible at one of the 3 hospital sites where maternity records were available.

We found higher rates of maternal and perinatal death in women with AKI than in those without. Small numbers of maternal deaths precluded further analysis of the strongest clinical predictors of this outcome; however, our data suggest that AKI may be a useful surrogate marker of maternal disease severity in preeclampsia, given the high rates of intensive care admission with increasing AKI severity. The finding of higher rates of perinatal death in women with AKI remained significant after adjustment for BP variables and gestation at admission, demonstrating that AKI is potentially an important independent predictor of poor outcome in the fetus, although further research with more detailed clinical data is required to validate this finding.

While equations used to calculate glomerular filtration rate are inaccurate in pregnancy,^16,17^ KDIGO AKI criteria have not been formally validated in pregnancy. KDIGO AKI criteria were chosen as this definition of AKI has gained international consensus.^18^ In calculating the KDIGO score, there were a small number of women (n=33) who only had raised creatinine concentrations of ≥90 μmol/L (≥1.02 mg/dL) on the National Laboratory databases. It is unknown whether these women had CKD or that KDIGO AKI criteria underestimated AKI severity of these women because of incomplete follow-up. Furthermore, preliminary identification of AKI by selection of the predefined outcome of maximum creatinine ≥90 µmol/L may have led to some cases of AKI being missed in women who had an increment in serum creatinine, which did not reach this threshold, although the renal implications of this are less clear.

### Comparison With Other Studies

The higher rates of maternal death and stillbirth associated with AKI in our study is in keeping with the findings of a recent systematic review and meta-analysis of pregnancy outcomes of pregnancy-related AKI.^6^ However, direct comparisons of incidence between studies is challenging because of differing definitions of pregnancy-related AKI.^5,19^ Only 1 study reported outcomes according to KDIGO AKI criteria.^11^ Maternal death is reported to occur in ≤20% of cases of pregnancy-related AKI, with dialysis requirements ranging from 0% to 54.6%^4,9,14^ with complete renal recovery in 69.4%,^5^ 89.4%,^7^ and 84.6%^9^ and dialysis dependence in 1.2%^5^ of cases. These diverse outcomes are likely to reflect different etiologies and definitions of AKI. The high rate of eclampsia within our cohort is in keeping with other published studies of preeclampsia from South Africa.^20^ The high rate of caesarean section is not unexpected as the majority of cases were of preterm preeclampsia, which is often associated with significant placental dysfunction which often precludes vaginal delivery.^21,22^

Established risk factors for AKI in nonpregnant populations include extremes of age, renal insufficiency, hypertension, diabetes mellitus, and cardiovascular disease.^23,24^ There is a paucity of studies investigating risk factors specifically for AKI within pregnancy. History of a previous pregnancy complicated by hypertension was identified to be associated with AKI in our study. The finding of a previous pregnancy complicated by hypertension as a risk factor for AKI was independent of other risk factors and was more common with increasing severity of AKI. If postadmission variables were also considered, maximal SBP alone was the best predictor of AKI and increasing severity of AKI. However, of note, the models had only modest predictive capacity (area under the receiver operator curve of 0.71 for combined model and 0.66 for maximum SBP alone). Susceptibility to AKI in patients with normal kidney function has recently been proposed to be increased in those with reduced renal reserve, that is, inability to augment glomerular filtration in response to protein loading.^25^ It is possible that a renal insult in a previous hypertensive pregnancy lowers functional reserve and contributes to reduced adaptive response in a subsequent pregnancy. Furthermore, preeclampsia has been reported to be 4-fold more common in women with a previous episode of AKI despite complete resolution of changes in serum creatinine.^26^ Thus, underlying subclinical renal disease could contribute to both susceptibility to recurrent preeclampsia and AKI.

In this study, the majority of women had resolution of AKI where follow-up data were available, with reducing rates of recovery with increasing AKI severity, which was also reported in a prospective Indian cohort study. However, incomplete renal recovery at 3 months postpartum was higher (9.5%) in the Indian cohort in which only 3.5% of women were lost to follow-up^4^ compared with incomplete renal recovery (2.6%) in our cohort. This may represent differing AKI severity or an underestimation of persistent renal injury in this study because of inadequate repeat sampling after discharge.

Outside of pregnancy, reported rates of recovery from AKI have varied between 33% and 90%, which is thought to be accounted for by different definitions of both AKI and renal recovery, differences in study populations, and differences in timing of assessment^27,28^ but in general appear to be lower than recovery rates for women with stages 2 and 3 AKI in our study (68.6% confirmed renal recovery). It is possible that pregnancy offers some protection against kidney injury, as suggested by recent work in animal models,^29^ and in addition, studies of AKI in pregnancy typically involve younger, healthier cohorts than studies of AKI in nonpregnant populations.

### Perspectives: Implications for Clinicians and Future Research

Awareness that recurrent preeclampsia in a second pregnancy may be associated with higher risk of AKI may be useful in triaging patients, frequency of creatinine concentration testing, and determining location of onward referral in low- and middle-income country settings. In addition, the association between maximum SBP and risk of AKI warrants further investigation including whether controlling BP in hypertensive pregnant women reduces incidence or severity of AKI and other maternal and perinatal morbidities. Further research is required to determine the short- and long-term outcomes of pregnancy-related AKI and whether this differs according to underlying pathogenesis. While currently, the outcomes of this condition are not fully understood, we would encourage clinicians to follow-up women who have experienced AKI who have not recovered renal function at discharge. The relationship between hypertensive disorders of pregnancy and renal reserve should be explored.

### Conclusions

Our study has identified that in a middle-income setting, pregnancy-related AKI complicates ≈15% of admissions with preeclampsia, and over half of these cases are severe (stage 2 or 3 AKI) with high rates of associated maternal (3.0%) and perinatal (37.1%) mortality. History of a previous hypertensive disorder of pregnancy was the most significant risk factor for development of AKI and worsening AKI severity. Approximately two-thirds of women had recovered from AKI at discharge, but further studies are required to determine the short- and long-term renal outcomes of pregnancy-related AKI.

## 

Acknowledgments

K. Bramham, F.I. Conti-Ramsden, L.C. Chappell, H.L. Nathan, and A.H. Shennan contributed to the conception. K. Bramham, F.I. Conti-Ramsden, L.C. Chappell, H.L. Nathan, and A.H. Shennan contributed to the design. F.I. Conti-Ramsden, A. De greeff, and D.R. Hall contributed to data acquisition. F.I. Conti-Ramsden, P.T. Seed, and K. Bramham contributed to analysis and interpretation. F.I. Conti-Ramsden and K. Bramham contributed to the input into drafting the article. F.I. Conti-Ramsden, K. Bramham, L.C. Chappell, P.T. Seed, H.L. Nathan, A.H. Shennan, A. De greeff, and D.R. Hall contributed to revision and final approval of the article.

## Sources of Funding

Funding for the project was provided by the Royal College of Obstetricians and Gynaecologists, the British Maternal Fetal Medicine Society, and the Medical Research Council/DFID/DBT Global Research Programme (MR/N006240/1). The original study (CRADLE 2) was funded by Bill and Melinda Gates Foundation (grant ID: OPP1086183).

## Disclosures

None.

## Supplementary Material

## Novelty and Significance

### What Is New?

- The novel finding of this study was the high proportion of women admitted with preeclampsia who developed acute kidney injury (AKI) that had a history of hypertensive disorder in a previous pregnancy, which was more frequent with increasing AKI severity.

### What Is Relevant?

- This study demonstrates the significant burden of morbidity of hypertensive disorders of pregnancy in middle-income settings; 15% of women admitted to hospital with preeclampsia had pregnancy-related AKI assessed by Kidney Disease Improving Global Outcomes criteria, with over half of cases being stage 2 or 3 in severity. Maternal death, eclampsia, stroke, and stillbirth rates were all higher in these women than those without.

### Summary

This study has identified that in a middle-income setting, pregnancy-related AKI complicates ≈15% of admissions with preeclampsia, and over half of these cases are severe (stage 2 or 3 AKI) with high rates of maternal (3.0%), and perinatal (37.1%) mortality. History of a previous hypertensive disorder of pregnancy was the most significant risk factor for development of AKI and worsening AKI severity. Approximately two-thirds of women had recovered from AKI at discharge, but further studies are required to determine the short-term and long-term renal outcomes of pregnancy-related AKI.

## References

[1] Hall DR, Conti-Ramsden F (2019). Acute kidney injury in pregnancy including renal disease diagnosed in pregnancy.. Best Pract Res Clin Obstet Gynaecol.

[2] Machado S, Figueiredo N, Borges A, São José Pais M, Freitas L, Moura P, Campos M (2012). Acute kidney injury in pregnancy: a clinical challenge.. J Nephrol.

[3] Stratta P, Besso L, Canavese C (1996). Is pregnancy-related acute renal failure a disappearing clinical entity?. Ren Fail.

[4] Stratta P, Canavese C, Dogliani M, Todros T, Gagliardi L, Vercellone A (1989). Pregnancy-related acute renal failure.. Clin Nephrol.

[5] Prakash J, Niwas SS, Parekh A, Pandey LK, Sharatchandra L, Arora P, Mahapatra AK (2010). Acute kidney injury in late pregnancy in developing countries.. Ren Fail.

[6] Liu Y, Ma X, Zheng J, Liu X, Yan T (2017). Pregnancy outcomes in patients with acute kidney injury during pregnancy: a systematic review and meta-analysis.. BMC Pregnancy Childbirth.

[7] Prakash J, Ganiger VC, Prakash S, Iqbal M, Kar DP, Singh U, Verma A (2018). Acute kidney injury in pregnancy with special reference to pregnancy-specific disorders: a hospital based study (2014-2016).. J Nephrol.

[8] Bucaloiu ID, Kirchner HL, Norfolk ER, Hartle JE, Perkins RM (2012). Increased risk of death and de novo chronic kidney disease following reversible acute kidney injury.. Kidney Int.

[9] Coca SG, Singanamala S, Parikh CR (2012). Chronic kidney disease after acute kidney injury: a systematic review and meta-analysis.. Kidney Int.

[10] Mills KT, Xu Y, Zhang W (2015). A systematic analysis of worldwide population-based data on the global burden of chronic kidney disease in 2010.. Kidney Int.

[11] Cooke WR, Hemmilä UK, Craik AL, Mandula CJ, Mvula P, Msusa A, Dreyer G, Evans R (2018). Incidence, aetiology and outcomes of obstetric-related acute kidney injury in Malawi: a prospective observational study.. BMC Nephrol.

[12] Fakhouri F, Vercel C, Frémeaux-Bacchi V (2012). Obstetric nephrology: AKI and thrombotic microangiopathies in pregnancy.. Clin J Am Soc Nephrol.

[13] Nathan HL, de Greeff A, Hezelgrave NL, Chappell LC, Shennan AH (2015). An accurate semiautomated oscillometric blood pressure device for use in pregnancy (including pre-eclampsia) in a low-income and middle-income country population: the Microlife 3AS1-2.. Blood Press Monit.

[14] Nathan HL, Seed PT, Hezelgrave NL, De Greeff A, Lawley E, Conti-Ramsden F, Anthony J, Steyn W, Hall DR, Chappell LC, Shennan AH (2018). Maternal and perinatal adverse outcomes in women with pre-eclampsia cared for at facility-level in South Africa: a prospective cohort study.. J Glob Health.

[15] Shema-Didi L, Ore L, Geron R, Kristal B (2010). Is anemia at hospital admission associated with in-hospital acute kidney injury occurrence?. Nephron Clin Pract.

[16] Alper AB, Yi Y, Webber LS, Pridjian G, Mumuney AA, Saade G, Morgan J, Nuwayhid B, Belfort M, Puschett J (2007). Estimation of glomerular filtration rate in preeclamptic patients.. Am J Perinatol.

[17] Smith MC, Moran P, Ward MK, Davison JM (2008). Assessment of glomerular filtration rate during pregnancy using the MDRD formula.. BJOG.

[18] Fujii T, Uchino S, Takinami M, Bellomo R (2014). Validation of the kidney disease improving global outcomes criteria for AKI and comparison of three criteria in hospitalized patients.. Clin J Am Soc Nephrol.

[19] Huang C, Chen S (2017). Acute kidney injury during pregnancy and puerperium: a retrospective study in a single center.. BMC Nephrol.

[20] Kenneth L, Hall DR, Gebhardt S, Grové D (2010). Late onset preeclampsia is not an innocuous condition.. Hypertens Pregnancy.

[21] Hall DR, Grové D, Carstens E (2006). Early pre-eclampsia: what proportion of women qualify for expectant management and if not, why not?. Eur J Obstet Gynecol Reprod Biol.

[22] van der Merwe JL, Hall DR, Wright C, Schubert P, Grové D (2010). Are early and late preeclampsia distinct subclasses of the disease–what does the placenta reveal?. Hypertens Pregnancy.

[23] Rewa O, Bagshaw SM (2014). Acute kidney injury-epidemiology, outcomes and economics.. Nat Rev Nephrol.

[24] Leblanc M, Kellum JA, Gibney RT, Lieberthal W, Tumlin J, Mehta R (2005). Risk factors for acute renal failure: inherent and modifiable risks.. Curr Opin Crit Care.

[25] Ronco C, Bellomo R, Kellum J (2017). Understanding renal functional reserve.. Intensive Care Med.

[26] Tangren JS, Adnan WAHWM, Powe CE (2018). Risk of preeclampsia and pregnancy complications in women with a history of acute kidney injury.. Hypertension.

[27] Forni LG, Darmon M, Ostermann M, Oudemans-van Straaten HM, Pettilä V, Prowle JR, Schetz M, Joannidis M (2017). Renal recovery after acute kidney injury.. Intensive Care Med.

[28] Kellum JA, Sileanu FE, Bihorac A, Hoste EA, Chawla LS (2017). Recovery after Acute Kidney Injury.. Am J Respir Crit Care Med.

[29] Popkov VA, Andrianova NV, Manskikh VN, Silachev DN, Pevzner IB, Zorova LD, Sukhikh GT, Plotnikov EY, Zorov DB (2018). Pregnancy protects the kidney from acute ischemic injury.. Sci Rep.

