# Supplementary figures and images for: Pregnancy-Related Acute Kidney Injury in Preeclampsia: Risk Factors and Renal Outcomes
Source: Hypertension. 2019 Sep 30;74(5):1144–51. doi: 10.1161/HYPERTENSIONAHA.119.13089 (PMC6791560; doi:10.1161/HYPERTENSIONAHA.119.13089)

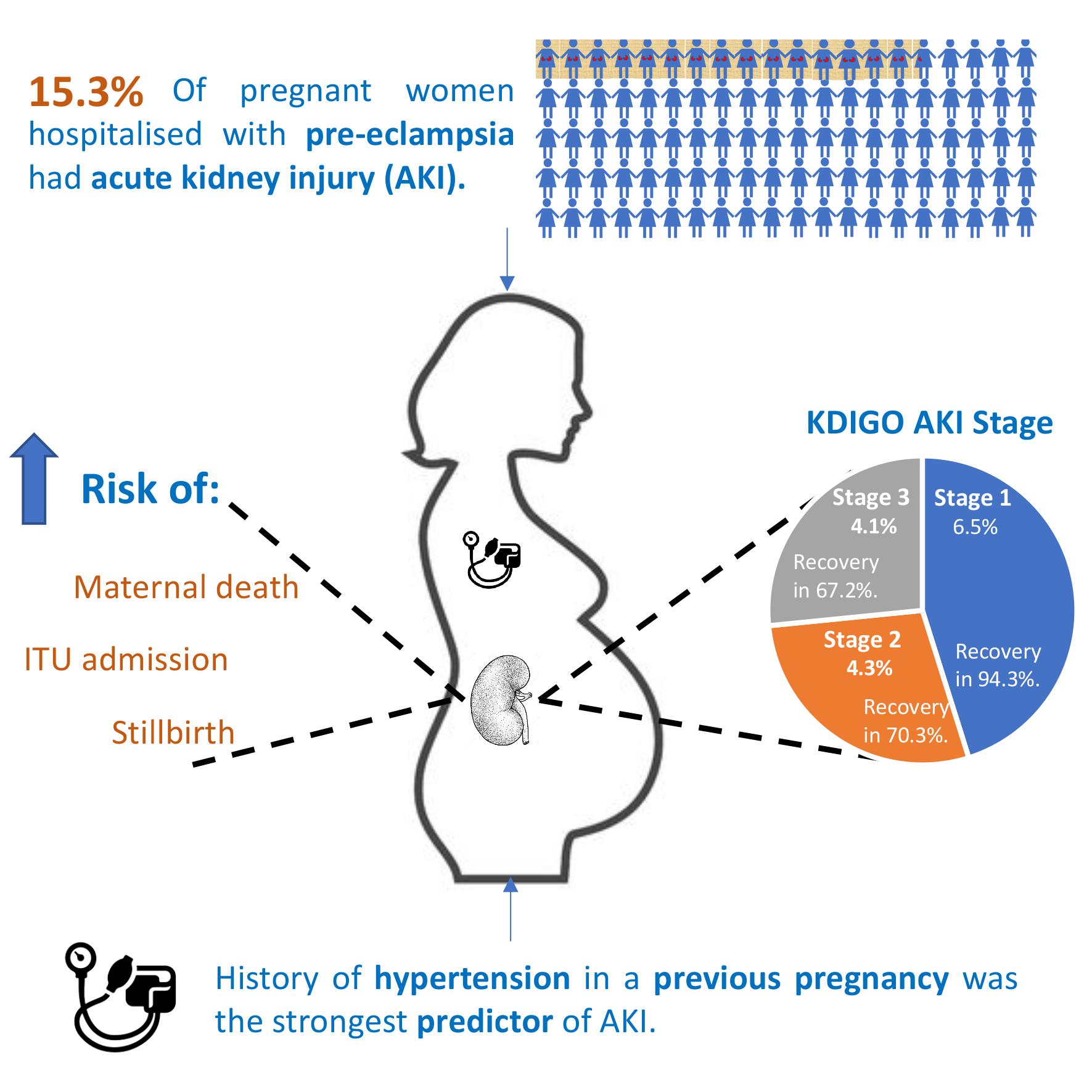

Supplement: Supplementary file 1 [file hyp-74-1144-s001.jpeg]
