# Supplementary material for: Pregnancy-Related Acute Kidney Injury in Preeclampsia: Risk Factors and Renal Outcomes
Source: Hypertension. 2019 Sep 30;74(5):1144–51. doi: 10.1161/HYPERTENSIONAHA.119.13089 (PMC6791560; doi:10.1161/HYPERTENSIONAHA.119.13089)
Supplement: Supplementary file 2 [file hyp-74-1144-s002.docx]

Supplementary Tables file for**:**

**Pregnancy-related Acute Kidney Injury in pre-eclampsia: risk factors and renal outcomes**

Running headline: Pr-AKI in pre-eclampsia

Frances I Conti-Ramsden MBBS MRCP^1^, Hannah L Nathan PhD^1^ , Annemarie De greeff^2^, David R Hall MMed MD^3^, Paul T Seed MSc CStat^1^, L C Chappell PhD FRCOG^1^, Andrew H Shennan MD FRCOG^1^, K Bramham MRCP, PhD^4^

**Affiliations:**

^1^: Department of Women and Children’s Health, King’s College London, London, UK
^2^: Accuracy Assessed Medical Devices CC, Diamond, South Africa
^3^: Stellenbosch University and Tygerberg Hospital, Cape Town, South Africa

^4^: Department of Renal Medicine, Division of Transplantation Immunology and Mucosal Biology, King’s College London, London, UK

**Corresponding author:**

Dr Frances Conti-Ramsden

Email: [fran.conti-ramsden@kcl.ac.uk](mailto:fran.conti-ramsden@kcl.ac.uk)

Address: Department of Women and Children’s Health, 10^th^ Floor North Wing, St Thomas’ Hospital, Westminster Bridge Road, London, SE1 7EH, UK.

Tel: 0207 188 3639

**Supplementary Tables**

**S1.** Haematology and biochemistry results at time of maximal creatinine in women with maximum creatinine (MaxCr) ≥ 90 μmol/L during admission with pre-eclampsia. Results presented as median (interquartile ratio).

| **Laboratory investigation** | **KDIGO AKI stage in women with MaxCr ≥ 90 μmol/L** | | | |  |
| --- | --- | --- | --- | --- | --- |
|  | AKI criteria not met n=35 | Stage 1  n=107 | Stage 2  n=67 | Stage 3  n=63 | p-value |
| **Max creatinine during admission (μmol/L)** | 101 (94-109) | 104 (94-122) | 118 (106-151) | 203 (133-455) | **<0.001** |
| **Haemoglobin (g/dL)** | 11.0 (9.4-12.3) | 10.7 (8.8-12.1) | 9.1 (8.0-10.9) | 8.7 (7.5-10.1) | **<0.001** |
| **White cell count (x10^9/L)** | 12.8 (9.9-16.3) | 13.3 (11.0-17.4) | 14.1 (11.5-17.4) | 17.6 (12.5-20.2) | **0.009** |
| **Platelets (x10^9/L)** | 154 (121-182) | 129 (75-209) | 121 (73-175) | 102 (67-155) | 0.178 |
| **Lactate dehydrogenase (U/L)** | 487 (407-522) | 550 (424-797) | 503 (387-736) | 689 (535-831) | **0.002** |
| **Sodium (mmol/L)** | 137 (135-140) | 136 (134-138) | 137 (134-139) | 134 (132-137) | **0.001** |
| **Potassium (mmol/L)** | 4.5 (4.1-4.7) | 4.3 (4.0-5.0) | 4.6 (4.1-4.9) | 4.3 (4.0-4.9) | 0.686 |
| **Urea (mmol/L)** | 5.9 (4.4-6.6) | 6.8 (5.3-8.0) | 7.0 (5.3-9.1) | 10.4 (8.0-18.9) | **<0.001** |
| **International normalized ratio** | 0.85 (0.83-0.94) | 0.91 (0.86-1.01) | 0.94 (0.86-1.06) | 0.94 (0.86-1.1)] | 0.623 |
| **Alanine aminotransferase (ALT) (U/L)** | 26 (16-81) | 31 (13-85) | 22 (15-53) | 41 (14-77) | 0.606 |
| **Aspartate transaminase (AST) (U/L)** | 43 (24-76) | 42 (26-113) | 42 (26-77) | 48 (34-92) | 0.431 |

**S2.** Maternal demographics and comorbidities in cases and controls by KDIGO AKI stage (5 categories). Results presented as no. (%).

| **Maternal demographics and comorbidities** | **No AKI** | | **AKI stage** | | |  |
| --- | --- | --- | --- | --- | --- | --- |
|  | Controls: MaxCr <90  (n=96) | MaxCr ≥90  (n=21) | Stage 1  (n=79) | Stage 2  (n=35) | Stage 3 (n=28) | p-value |
| ***Demographics*** |  |  |  |  |  |  |
| **Age**  <20  20-29  30-39  >=40 | 14 (14.6)  50 (52.1)  31 (32.3)  1 (1.0) | 4 (19.0)  12 (57.1)  4 (19.0)  1 (4.8) | 4 (5.1)  35 (44.3)  34 (43.0)  6 (7.6) | 2 (5.7)  15 (42.9)  18 (51.4)  0 (0.0) | 3 (10.7)  11 (39.3)  14 (50.0)  0 (0.0) | **0.008** |
| **BMI**  18.5-24.9  25-29.9  30-34.9  >=35 | 20 (20.8)  23 (24.0)  23 (24.0)  30 (31.3) | 11 (52.4)  6 (28.6)  0 (0.0)  4 (19.0) | 25 (31.6)  20 (25.3)  18 (22.8)  16 (20.3) | 7 (20.0)  8 (22.9)  7 (20.0)  13 (37.1) | 10 (35.7)  7 (25.0)  3 (10.7)  8 (28.6) | 0.381 |
| ***Medical history*** |  |  |  |  |  |  |
| **Chronic Hypertension** | 18 (18.8) | 0 (0.0) | 20 (25.3) | 10 (28.6) | 11 (39.3) | **0.011** |
| **Anaemia**  (Hb <9g/dL) | 5 (5.2) | 2 (9.5) | 3 (3.8) | 3 (8.6) | 2 (7.1) | 0.691 |
| **HIV** | 17 (17.7) | 0 (0.0) | 6 (7.6) | 4 (11.4) | 10 (35.7) | 0.341 |
| ***Pregnancy history*** |  |  |  |  |  |  |
| **Primiparity** | 37 (38.5) | 12 (57.1) | 26 (32.9) | 10 (28.6) | 8 (28.6) | 0.142 |
| **Previous HDP** | 17 (17.7) | 3 (14.3) | 20 (25.3) | 12 (34.3) | 11 (39.3) | **0.015** |

MaxCr: maximum creatinine concentration in μmol/L.

HDP: hypertensive disorder of pregnancy.
